# Perfluorooctane Sulfonate Concentrations in Amniotic Fluid, Biomarkers of Fetal Leydig Cell Function, and Cryptorchidism and Hypospadias in Danish Boys (1980–1996)

**DOI:** 10.1289/ehp.1409288

**Published:** 2015-06-05

**Authors:** Gunnar Toft, Bo A.G. Jönsson, Jens Peter Bonde, Bent Nørgaard-Pedersen, David M. Hougaard, Arieh Cohen, Christian H. Lindh, Richard Ivell, Ravinder Anand-Ivell, Morten S. Lindhard

**Affiliations:** 1 Department of Occupational Medicine, and; 2 Department of Clinical Epidemiology, Aarhus University Hospital, Aarhus, Denmark; 3 Division of Occupational and Environmental Medicine, Lund University, Lund, Sweden; 4 Department of Occupational and Environmental Medicine, Copenhagen University Hospital, Bispebjerg, Copenhagen, Denmark; 5 Danish Center for Neonatal Screening, Department of Clinical Biochemistry and Immunology, Statens Serum Institute, Copenhagen, Denmark; 6 School of Biosciences, University of Nottingham, Nottingham, United Kingdom; 7 Department of Pediatrics, Regional Hospital of Randers, Randers, Denmark; 8 Perinatal Epidemiology Research Unit, Department of Pediatrics, Aarhus University Hospital, Skejby, Denmark

## Abstract

**Background:**

Exposure to perfluorooctane sulfonate (PFOS) may potentially disturb fetal Leydig cell hormone production and male genital development.

**Objectives:**

We aimed to study the associations between levels of amniotic fluid PFOS, fetal steroid hormone, and insulin-like factor 3 (INSL3) and the prevalence of cryptorchidism and hypospadias.

**Methods:**

Using the Danish National Patient Registry, we selected 270 cryptorchidism cases, 75 hypospadias cases, and 300 controls with stored maternal amniotic fluid samples available in a Danish pregnancy-screening biobank (1980–1996). We used mass spectrometry to measure PFOS in amniotic fluid from 645 persons and steroid hormones in samples from 545 persons. INSL3 was measured by immunoassay from 475 persons. Associations between PFOS concentration in amniotic fluid, hormone levels, and genital malformations were assessed by confounder-adjusted linear and logistic regression.

**Results:**

The highest tertile of PFOS exposure (> 1.4 ng/mL) in amniotic fluid was associated with a 40% (95% CI: –69, –11%) lower INSL3 level and an 18% (95% CI: 7, 29%) higher testosterone level compared with the lowest tertile (< 0.8 ng/mL). Amniotic fluid PFOS concentration was not associated with cryptorchidism or hypospadias.

**Conclusions:**

Environmental PFOS exposure was associated with steroid hormone and INSL3 concentrations in amniotic fluid, but was not associated with cryptorchidism or hypospadias in our study population. Additional studies are needed to determine whether associations with fetal hormone levels may have long-term implications for reproductive health.

**Citation:**

Toft G, Jönsson BA, Bonde JP, Nørgaard-Pedersen B, Hougaard DM, Cohen A, Lindh CH, Ivell R, Anand-Ivell R, Lindhard MS. 2016. Perfluorooctane sulfonate concentrations in amniotic fluid, biomarkers of fetal Leydig cell function, and cryptorchidism and hypospadias in Danish boys (1980–1996). Environ Health Perspect 124:151–156; http://dx.doi.org/10.1289/ehp.1409288

## Introduction

Perfluorooctane sulfonate (PFOS) has until recently been widely used in a variety of applications, especially in surface coatings used to make products water- and oil-resistant. PFOS is highly biopersistent with a half-life in humans of about 5 years (Olsen et al. 2007). PFOS was added to Annex B of the Stockholm Convention on Persistent Organochlorine Pollutants in 2009, and the production and use of PFOS have been regulated in Europe since 2008 (European Commission 2006). In Norway, PFOS levels rose through the mid-1990s, and have declined since 2000 (Haug et al. 2009). However, model-based estimates of future environmental PFOS exposures suggest a slower decay in temperate regions (Armitage et al. 2009). PFOS has been detected in human populations from all over the world, but considerable variation in exposure has been observed between populations (Lau et al. 2004). Most previous studies have measured PFOS in serum, and the highest concentrations have been observed among workers at facilities producing PFOS, with mean PFOS concentrations of 1,000–2,000 ng/mL, whereas general populations on average had concentrations of about 35 ng/mL PFOS around the peak exposure period (Lau et al. 2004). Apart from the present study population (Jensen et al. 2012), PFOS has to our knowledge been measured in amniotic fluid only in one recent American study of 28 women (Stein et al. 2012). That study indicated a PFOS concentration in amniotic fluid about 20-fold less than in maternal serum.

Experimental studies of the effects of PFOS on adult male reproductive function have reported reduced testosterone production, altered gonadotropin secretion, and reduced epididymal sperm in PFOS-treated rats and mice (López-Doval et al. 2014; Wan et al. 2011). Studies of human adults have shown associations between PFOS exposure and the proportion of normal sperm cells (Joensen et al. 2009; Toft et al. 2012) although these results were not corroborated in a recent study (Joensen et al. 2013), and other aspects of semen quality seem to be unaffected (Joensen et al. 2009, 2013; Toft et al. 2012). Apart from an inverse association between PFOS exposure and testosterone level in one study (Joensen et al. 2013), no significant associations between PFOS exposure and reproductive hormones in adult men have been observed (Joensen et al. 2009; Raymer et al. 2012; Specht et al. 2012).

There have been fewer studies on the effects of PFOS exposure on fetal steroidogenesis, and later reproductive function. A study on rat fetal Leydig cell function indicated that 20 mg/kg/day exposure of pregnant rats from gestational days 11 to 19 was associated with reduced testosterone production, reduced fetal Leydig cell number, and decreased expression of steroidogenic enzymes (Zhao et al. 2014). Also, zebrafish embryos showed altered expression of stereoidogenic enzymes after exposure to up to 500 μg/L PFOS from 4 to 120 hr past fertilization (Du et al. 2013). Vested et al. (2013) studied whether prenatal exposure to PFOS (measured in maternal serum) was associated with adult male reproductive function among 169 Danish men born in 1988–1989. Although prenatal PFOS exposure was not associated with reproductive hormones or semen quality, prenatal exposure to perfluorooctanoate (PFOA) was associated with reduced semen quality and higher levels of luteinizing hormone (LH) and follicle-stimulating hormone (FSH) in young adult men. These results suggest that the prenatal period may be sensitive to environmental exposures to perfluoroalkyl substances (PFAS).

Evidence of effects on fetal Leydig cell hormone production has led others to hypothesize that PFOS exposure might affect androgen- and insulin-like factor 3 (INSL3)–dependent testicular descent (Bay et al. 2011) and scrotal fusion (Kalfa et al. 2009). Only one previous study has evaluated the risk of cryptorchidism in relation to PFAS exposure; this study did not observe associations between PFAS level in cord blood and cryptorchidism (Vesterholm et al. 2014). However, due to a limited study size of 59 cryptorchidism cases and 108 matched controls, the power to show an association was limited. To our knowledge, no previous studies have evaluated the potential association between *in utero* exposure to PFOS and fetal hormone level or hypospadias.

The aim of the present study was to determine whether *in utero* PFOS levels are associated with altered Leydig cell function, as indicated by steroid hormone and INSL3 concentrations in amniotic fluid, and to evaluate whether these potential effects on hormone levels were associated with genital malformation in offspring.

## Methods

*Study population and amniotic fluid samples*. The study population has been described previously in detail (Jensen et al. 2012). Briefly, we used amniotic fluid samples from a Danish biobank maintained at the State Serum Institute in Copenhagen (http://www.ssi.dk). The biobank holds samples from a pregnancy-screening registry, including information on amniotic fluid samples from 63,882 pregnancies covering the period 1980–1996. After restriction to live-born singleton boys with complete obstetric data, 25,105 pregnancies were eligible (Jensen et al. 2012). The amniotic fluid samples were centrifuged before routine diagnostic analyses, and the supernatants were kept frozen at –20°C until the present analyses were carried out. Indications for amniocentesis included age ≥ 35 years and/or increased risk of severe malformations or Down syndrome based on results from maternal serum analyses. We calculated the gestational week of amniocentesis based on the date of amniocentesis, the date of birth, and the estimated gestational age at birth as described by Jensen et al. (2012). Each sample in the pregnancy-screening registry was recorded by the personal identification number (unique to each Danish citizen) of the pregnant woman. We used these unique identifiers to obtain obstetric data on the pregnancies from the Danish Medical Birth Registry (Knudsen and Olsen 1998), including gestational age at birth, singleton or multiple birth, maternal parity, and birth weight and Apgar score of the infant. In addition, we used the unique identifiers in the Danish Civil Registration System to identify male offspring from the pregnancies (Pedersen et al. 2006).

The Danish Regional Ethics Committee, the Danish National Board of Health, and the Danish Data Protection Agency approved the study. The use of the biobank for research purposes has been approved, and additional informed consent from the study subjects for this specific project was neither recommended nor required.

*Case–control definitions and ascertainment*. Controls were randomly selected from the 25,105 amniotic fluid samples belonging to live-born male offspring pregnancies in the screening database with complete obstetric data in the Danish Medical Birth Registry. The number of controls (*n* = 412) was chosen to roughly equal the largest case group consisting of 404 boys with cryptorchidism. Cryptorchidism cases had both a diagnosis of undescended testis according to the *International Classification of Diseases, 8th and 10th Revisions* [ICD-8: 75210, 75211, 75219; ICD-10: Q53, Q531(A), Q532(A), Q539] and a corrective surgical procedure according to the Surgery and Treatment Classification of the Danish National Board of Health (STC: 55600, 55640) or the Nordic Classification of Surgical Procedures (NCSP: KKFH00, KKFH01, KKFH10) recorded in the Danish National Patient Registry (DNPR). Boys with a registry entry of inguinal hernia repair (STC: 40620, 40640; NCSP: KJAB00-KJAB97) were excluded from this case group to avoid iatrogenic cryptorchidism secondary to hernia repair. We included all boys that fulfilled these criteria (404 of 25,105; 1.61%) to maximize the cryptorchidism case group and overall study size. For the hypospadias case group we included all 109 of the 25,105 boys (0.43%) with a diagnosis of hypospadias in the DNPR (ICD-8: 75220, 75221, 75222, 75228, 75229; ICD-10: Q540, Q541, Q542, Q548, Q549). All boys were followed for the aforementioned diagnoses and surgery entries in the DNPR from birth until November 2008.

*Measurement of chemical compounds and hormones*. During all chemical analyses, laboratory technicians were blinded to case–control status and to levels of analytes measured by others. We assayed PFOS and cotinine in amniotic fluid as described in detail by Jensen et al. (2012). Coefficients of variation were 11% for PFOS and 9% for cotinine; the limit of detection (LOD) was 0.20 ng/mL for PFOS and cotinine, determined as the concentrations corresponding to three times the standard deviation of the responses in chemical blanks. We analyzed the samples using a liquid chromatograph (LC; model UFLCXR, Shimadzu Corp). The LC was connected to a hybrid triple quadrupole linear ion trap tandem mass spectrometer (LC/MS/MS) equipped with a turbo ion spray source (QTRAP 5500; AB Sciex).

We assayed the steroid hormones testosterone, androstenedione, progesterone, 17-OH-progesterone, and cortisol in amniotic fluid at the Danish Center for Neonatal Screening, Department of Clinical Biochemistry and Immunology, the State Serum Institute, Copenhagen, Denmark. The online extraction LC-MS system consisted of an Aria TLX2 system (Thermo Scientific) with two Agilent 1100 binary pumps and two Agilent 1200 quaternary pumps (Agilent) connected to a Thermo TSQ Ultra triple quadrupole mass spectrometer equipped with an APCI ion source. Extraction was performed using a Cyclone P 0.5 × 50 mm Turboflow column (Thermo Scientific), and analytical separation was achieved using Kinetix 2.6 μ 2.1 × 50 mm C18 columns (Phenomenex). All calibrators, controls, internal standards, and micro-titer plates were purchased from PerkinElmer via their CHS steroid profiling kit for mass spectrometry (PerkinElmer). Formic acid was purchased from Merck. Ammonium acetate and zinc sulphate heptahydrate were purchased from Sigma-Aldrich. Water was purified using a water purification unit from Millipore. We used a volume of 50 μL amniotic fluid for the assay. The LODs and the intra- and interassay coefficients of variation were as follows: testosterone, 0.1 nmol/L, 9 and 10%, respectively; androstenedione, 0.3 nmol/L, 8 and 9%, respectively; progesterone, 0.4 nmol/L, 10 and 11%, respectively; 17-OH-progesterone, 0.4 nmol/L, 9 and 10%, respectively; and cortisol, 4.4 nmol/L, 10 and 12%, respectively.

INSL3 in amniotic fluid was measured using a semicompetitive time-resolved fluorometric immunoassay (TRFIA) slightly modified from that described in detail by Anand-Ivell et al. (2006). The laboratory work was performed at Leibniz Institute for Farm Animal Biology, Dummerstorf, Germany. The modifications involved the replacement of the original rat antiserum by a new polyclonal antiserum (no. #RIA5) raised in rabbits (IMVS Antibody Services) against the same chemically synthesized human INSL3 as previously, at a final dilution of 1:10,000. As tracer, we used the same Europium-labeled human INSL3 as described (Anand-Ivell et al. 2006). Accordingly, also the secondary goat-anti-rabbit antibody (Rockland Immunochemicals Inc.) used to coat the plates substituted for the original anti-rat antibody. All other conditions were similar. Standard curves for measurement of amniotic fluid were constructed using serial dilutions of human INSL3 in EDTA–phosphate-buffered saline (Anand-Ivell et al. 2008). A volume of 100 μL amniotic fluid was used for the assay, and the limit of detection for the modified assay was 0.01 ng/mL, with inter- and intraplate coefficients of variation of < 8% and < 1%, respectively, across the range. There was no cross-reactivity detectable across the physiological range with the structurally related peptides, insulin, IGF-1 (insulin-like growth factor-1), and relaxin. There was also no cross-reactivity detectable with rat INSL3, and only 10% cross-reactivity with bovine INSL3 at the highest values. Spiking experiments into normal male and female human sera from previous studies (Anand-Ivell et al. 2006, 2013) indicated 122.9 + 1.5% and 96.5 + 2.7% recovery, respectively. INSL3 measurements of human serum following five successive freeze–thaw cycles showed no significant change (data not shown).

*Statistical analysis*. We imputed values below the LOD of our chemical assays with a random value between the LOD and LOD/2 as a simplified method of maintaining variability in values below LOD. For the INSL3 assay, 58 of 543 (10.7%) samples were below the LOD (0.01 ng/mL). For the PFOS assay, 10 of 645 (1.6%) samples were below the LOD (0.2 ng/mL). For the steroid assay (*n* = 574), we used the instrument readout for values below the LOD; two (0.3%) testosterone values < 0.1 nmol/L, no androstenedione values < 0.3 nmol/L, no progesterone values < 0.4 nmol/L, no 17-OH-progesterone values < 0.4 nmol/L, and six (1.0%) cortisol values < 4.4 nmol/L.

INSL3 levels are highly dependent on gestational age at amniocentesis with a peak around week 15 (Anand-Ivell et al. 2008). We calculated multiple of the median (MoM) values for INSL3 by gestational age at amniocentesis to reduce this dependence (Knight and Palomaki 2003). We used the random sample control group to estimate the median level for each week, and all INSL3 values (cases and controls) were then divided by the median of the corresponding week to calculate the MoM value. Weeks 11–13 were pooled because of limited number of observations, and the median value from week 21 (controls) was applied to week 22 (cases) because there were no controls in week 22. All statistical analyses were presented for both the raw and the MoM INSL3 measures.

Associations between PFOS exposure and the included outcomes were evaluated by multiple linear or logistic regression analyses for continuous and categorical outcomes, respectively. We divided PFOS exposure into tertiles to quantify the difference between the highest and lowest third of the population and to evaluate whether any marked deviations from a linear association was evident.

Differences in median hormone level between the PFOS tertiles were evaluated by the nonparametric Kruskal–Wallis test. Then adjusted linear regression analyses were performed evaluating tertile differences. We additionally performed regression analyses on a continuous PFOS variable to test for a linear trend.

Data are presented as difference from the lowest tertile and β [95% confidence interval (CI)] for linear regression models both unadjusted and adjusted. We performed interaction analyses including a ln-PFOS × case/control group term in the regression models to evaluate whether combined analyses of cases of cryptorchidism, hypospadias, and control groups were statistically justifiable. In supplementary analyses, we additionally evaluated the association between PFOS and hormones separately for controls, cryptorchidism cases, and hypospadias cases to evaluate whether the association varied across groups. We *a priori* decided to adjust all hormone analyses for gestational age of amniocentesis (continuous, weeks), maternal age (years), and smoking (using amniotic fluid specific levels as a biomarker for nonsmoker: < 25 ng/mL cotinine; passive smoker: 25–85 ng/mL; and smoker: > 85 ng/mL) (Jauniaux et al. 1999). Analysis of combined associations between PFOS and hormone levels across case and control groups were additionally adjusted for case–control status. The logistic regression analyses of cryptorchidism and hypospadias was adjusted for gestational age of amniocentesis (continuous, weeks), year of amniocentesis (three groups), maternal age (years), gestational age (weeks), birth weight (grams), and smoking (cotinine groups). To further evaluate whether year of amniocentesis influenced these associations, we made supplementary stratified analysis by year of amniocentesis (1980–1986, 1987–1992, and 1992–1996).

We also restricted the analyses to boys with no other congenital malformations to exclude cases with syndromes and chromosomal abnormalities.

PFOS and all hormone data were transformed by the natural logarithm (ln) to improve normality of their distribution of residuals in the regression analyses.

## Results

Of the original 925 included subjects in the cohort, we could not locate samples in the biobank for 60 subjects, and 220 had insufficient volume for PFOS measurements. Thus, the study population consists of 645 mother–child pairs with information on fetal PFOS exposure and case status of the children, including 270 cases of cryptorchidism, 75 cases of hypospadias, and a random control group of 300. The mean and standard deviation of maternal characteristics including age and gestational age of amniocentesis and distribution of smoking prevalence did not differ markedly between the case and control groups (Table 1). As expected, the cryptorchidism and hypospadias cases weighed less and were on average born slightly earlier than the control group. Also, the distribution of calendar year of amniocentesis differed between case groups (Table 1).

**Table 1:** Characteristics of the included pregnancies and boys by case–control status among Danish pregnant women with amniocentesis (1980–1996).

Characteristics	Control (*n* = 300)	Cryptorchidism (*n* = 270)	Hypospadias (*n* = 75)
Maternal age at birth [years (mean ± SD)]	32.6 ± 5.3	32.8 ± 5.3	31.3 ± 5.8
Gestational week of amniocentesis (mean ± SD)	15.7 ± 1.3	15.9 ± 1.8	15.6 ± 1.6
Gestational age at birth [weeks (mean ± SD)]	39.6 ± 1.5	39.2 ± 2.2	38.7 ± 2.8
Birth weight [g (mean ± SD)]	3,535 ± 561	3,370 ± 682	3,216 ± 827
Year of amniocentesis (%)
1980–1984	34	40	17
1985–1990	34	32	37
1991–1996	33	29	45
Smoking (%)
Nonsmoker: cotinine < 25 ng/mL	61	60	58
Passive smoker: cotinine 25–85 ng/mL	6	4	7
Smoker: cotinine ≥ 85 ng/mL	33	36	35
Congentital malformations (%)^*a*^	6	14	20
^***a***^Malformations other than cryptorchidism and hypospadias.			

Amniotic fluid steroid hormone levels were measured on 545 pregnant women with sufficient sample volume (84% of cryptorchidism cases, 100% of hypospadias cases, and 81% of control samples). Testosterone level was positively associated with PFOS exposure with 18% higher testosterone (95% CI: 7, 29%) in the highest PFOS tertile compared with the lowest in the overall population (all three groups combined). Also, the linear trend test showed a significant positive association in both the unadjusted and adjusted analysis (Table 2).

**Table 2:** PFOS exposure (ng/mL) and markers of Leydig cell function in amniotic fluid among Danish pregnant women with amniocentesis (1980–1996).

Hormone and PFOS exposure	*n*	Median hormone level	% difference (95% CI)^*a*^
Testosterone (nmol/L)	545
< 0.8 ng/mL PFOS		0.73	Reference
0.8–1.4 ng/mL PFOS		0.81	9 (–2, 20)
> 1.4 ng/mL PFOS		0.91	18 (7, 29)
*p*-Value^*b*^		0.002
Ln-PFOS (crude)^*c*^			0.14 (0.08, 0.22)
Ln-PFOS (adjusted)^*c*^			0.16 (0.09, 0.23)
*p*-Value for interaction^*d*^			0.68
DHEAS (nmol/L)	545
< 0.8 ng/mL PFOS		17.0	Reference
0.8–1.4 ng/mL PFOS		17.4	5 (–10, 20)
> 1.4 ng/mL PFOS		17.4	2 (–14, 17)
*p*-Value^*b*^		0.93	
Ln-PFOS (crude)^*c*^			0.06 (–0.03, 0.16)
Ln-PFOS (adjusted)^*c*^			0.07 (–0.03, 0.16)
*p*-Value for interaction^*d*^			0.03
Androstendione (nmol/L)	545
< 0.8 ng/mL PFOS		2.62	Reference
0.8–1.4 ng/mL PFOS		2.72	8 (0, 17)
> 1.4 ng/mL PFOS		3.07	17 (8, 25)
*p*-Value^*b*^		0.001
Ln-PFOS (crude)^*c*^			0.15 (0.10, 0.20)
Ln-PFOS (adjusted)^*c*^			0.15 (0.10, 0.21)
*p*-Value for interaction^*d*^			0.22
Progesterone (nmol/L)	545
< 0.8 ng/mL PFOS		163.7	Reference
0.8–1.4 ng/mL PFOS		170.8	11 (0, 23)
> 1.4 ng/mL PFOS		186.4	22 (11, 34)
*p*-Value^*b*^		0.001
Ln-PFOS (crude)^*c*^			0.21 (0.14, 0.28)
Ln-PFOS (adjusted)^*c*^			0.21 (0.14, 0.29)
*p*-Value for interaction^*d*^			0.07
17-OH-Progesterone (nmol/L)	545
< 0.8 ng/mL PFOS		4.94	Reference
0.8–1.4 ng/mL PFOS		5.22	7 (–1, 13)
> 1.4 ng/mL PFOS		6.18	18 (11, 26)
*p*-Value^*b*^		< 0.001
Ln-PFOS (crude)^*c*^			0.17 (0.12, 0.22)
Ln-PFOS (adjusted)^*c*^			0.15 (0.11, 0.20)
*p*-Value for interaction^*d*^			0.92
Cortisol (nmol/L)	545
< 0.8 ng/mL PFOS		15.9	Reference
0.8–1.4 ng/mL PFOS		17.0	9 (–0, 17)
> 1.4 ng/mL PFOS		22.4	28 (19, 37)
*p*-Value^*b*^		< 0.001
Ln-PFOS (crude)^*c*^			0.26 (0.20, 0.32)
Ln-PFOS (adjusted)^*c*^			0.19 (0.13, 0.25)
*p*-Value for interaction^*d*^			0.96
INSL3 (ng/mL)	475
< 0.8 ng/mL PFOS		0.14	Reference
0.8–1.4 ng/mL PFOS		0.12	–21 (–48, 7)
> 1.4 ng/mL PFOS		0.09	–40 (–69,–11)
*p*-Value^*b*^		< 0.001
Ln-PFOS (crude)^*c*^			–0.35 (–0.54, –0.16)
Ln-PFOS (adjusted)^*c*^			–0.21 (–0.40, –0.02)
*p*-Value for interaction^*d*^			0.98
INSL3 (MoM)^*e*^	475
< 0.8 ng/mL PFOS		1.45	Reference
0.8–1.4 ng/mL PFOS		1.13	–21(–49, 8)
> 1.4 ng/mL PFOS		0.85	–39 (–68,–10)
*p*-Value^*b*^		0.07
Ln-PFOS (crude)^*c*^			–0.17 (–0.36, 0.01)
Ln-PFOS (adjusted)^*c*^			–0.20 (–0.38, –0.01)
*p*-Value for interaction^*d*^			0.82
^***a***^Adjusted for gestational age of amniocentesis, maternal age, smoking (cotinine groups), and case or control status unless otherwise indicated. ^***b***^Kruskal–Wallis rank test. ^***c***^Estimates represent the percent difference in hormone concentrations with a 1% increase in PFOS. ^***d***^Interaction terms (lnPFOS × case–control status) in adjusted linear regression models. ^***e***^Multiple of the median (MoM) values are calculated as specified in the methods.			

Also, androstendione, progesterone, 17-OH-progesterone, and cortisol, but not DHEAS (dehydroepiandrosterone sulfate), were positively associated with PFOS exposure (Table 2). INSL3 was measured in amniotic fluid samples from 475 pregnancies (74% of cryptorchidism cases, 84% of hypospadias cases, and 71% of controls). INSL3 was negatively associated with PFOS, with a 40% lower INSL3 concentration (95% CI: –69, –11%) estimated for the highest tertile compared with the lowest based on the overall adjusted model, and approximately the same magnitude when evaluated as INSL3 MoM. The inverse associations were confirmed by linear trend analyses (Table 2).

The association between PFOS exposure, testosterone, and INSL3 was similar in the case and control groups as indicated by *p*-values of product interaction terms in the regression models (Table 2) and estimates (Table 3). However, in the stratified analysis, fewer statistical significant associations were observed, probably because of a lower number of cases (Table 3).

**Table 3:** PFOS exposure (ng/mL) and markers of Leydig cell function in amniotic fluid among Danish pregnant women with amniocentesis (1980–1996) stratified by case–control group.

Hormone and PFOS exposure	Controls		Cryptorchism		Hypospadias	
	*n*	% difference (95% CI)^*a*^	*n*	% difference (95% CI)^*a*^	*n*	% difference (95% CI)^*a*^
Testosterone (nmol/L)	242		228		75
< 0.8 ng/mL PFOS		Reference		Reference		Reference
0.8–1.4 ng/mL PFOS		10 (–5, 24)		14 (–4, 32)		–12 (–47, 24)
> 1.4 ng/mL PFOS		19 (4, 33)		20 (3, 38)		–6 (–47, 36)
Ln-PFOS (crude)^*b*^		0.12 (0.03, 0.21)		0.18 (0.08, 0.28)		0.13 (–0.11, 0.37)
Ln-PFOS (adjusted)^*b*^		0.14 (0.04, 0.23)		0.20 (0.09, 0.31)		0.00 (–0.28, 0.28)
INSL3	213		199		63
< 0.8 ng/mL PFOS		Reference		Reference		Reference
0.8–1.4 ng/mL PFOS		–28 (–72, 16)		–5 (–47, 38)		–49 (–118, 20)
> 1.4 ng/mL PFOS		–37 (–81, 8)		–38 (–82, 6)		–21 (–110, 69)
Ln-PFOS (crude)^*b*^		–0.26 (–0.54, 0.02)		–0.39 (–0.68, –0.11)		–0.62 (–1.23, 0.00)
Ln-PFOS (adjusted)^*b*^		–0.22 (–0.51, 0.06)		–0.20 (–0.48, 0.07)		0.02 (–0.60, 0.64)
INSL3 MoM^*c*^	213		199		63
< 0.8 ng/mL PFOS		Reference		Reference		Reference
0.8–1.4 ng/mL PFOS		–30 (–73,12)		0 (–44, 45)		–60 (–135, 15)
> 1.4 ng/mL PFOS		–43 (–86, –0)		–30 (–75, 15)		–20 (–117, 77)
Ln-PFOS (crude)^*b*^		–0.24 (–0.51, 0.03)		–0.09 (–0.37, 0.18)		–0.28 (–0.87, 0.30)
Ln-PFOS (adjusted)^*b*^		–0.27 (0.54, 0.00)		–0.12 (–0.40, 0.17)		–0.01 (–0.68, 0.65)
^***a***^Adjusted for gestational age of amniocentesis, maternal age, smoking (cotinine groups), and case or control status unless otherwise indicated. ^***b***^Estimates represent the percent difference in hormone concentrations with a 1% increase in PFOS. ^***c***^Multiple of the median (MoM) values are calculated as specified in the methods.						

PFOS concentrations in amniotic fluid were not associated with cryptorchidism or hypospadias based on adjusted logistic regression models (Table 4). Supplementary analysis stratifying these associations by sampling year (1980–1986, 1987–1992, and 1992–1996) also did not indicate any association between PFOS exposure and odds for cryptorchidism or hypospadias (data not shown). Furthermore, analyses restricted to boys with no other congenital malformations (numbers given in Table 1) produced essentially unchanged results (data not shown).

**Table 4:** Odds ratios (95% CI) for cryptorchidism or hypospadias in relation to amniotic fluid PFOS concentration among Danish pregnant women with amniocentesis (1980–1996).

Malformation	*n* control	*n* cases	Unadjusted OR (95% CI)	Adjusted OR (95% CI)^*a*^
Cryptorchidism
1st tertile	102	86	1.0 (reference)	1.0 (reference)
2nd tertile PFOS	101	91	1.11 (0.74, 1.67)	1.08 (0.71, 1.63)
3rd tertile PFOS	97	93	1.09 (0.73, 1.63)	1.01 (0.66, 1.53)
Ln-PFOS	300	270	1.05 (0.81, 1.35)	0.99 (0.75, 1.30)
Hypospadias
1st tertile	102	27	1.0 (reference)	1.0 (reference)
2nd tertile PFOS	101	26	1.01 (0.55, 1.81)	0.97 (0.51, 1.87)
3rd tertile PFOS	97	22	0.82 (0.44, 1.54)	0.69 (0.35, 1.38)
Ln-PFOS	300	75	0.97 (0.65, 1.44)	0.87 (0.57, 1.34)
^***a***^Adjusted for gestational age of amniocentesis, year of amniocentesis (groups), maternal age, gestational age, birth weight, and smoking (cotinine groups).				

## Discussion

PFOS concentrations measured in amniotic fluid were associated with higher steroid hormone levels and lower INSL3 in the combined study population but were not associated with cryptorchidism or hypospadias (270 and 75 cases, respectively, compared with 300 controls).

Our results are consistent with those of Vesterholm et al. (2014), who reported no association of cord blood concentrations of PFOS or other PFAS with cryptochidism in 215 Danish and Finnish boys.

To our knowledge, the present study is the first human study evaluating potential association between fetal exposure to PFOS and biomarkers of human fetal steroid and INSL3 levels. A study using the human adrenocortical carcinoma (H295R) *in vitro* cell assay reported increased testosterone, progesterone, and estradiol secretion after PFOS exposure (Kraugerud et al. 2011), supporting the possibility that prenatal PFOS exposure may increase fetal steroid hormone production.

The inverse association between prenatal concentrations of PFOS and INSL3 may be consistent with a direct effect on fetal Leydig cells, because, based on the current knowledge, INSL3 is produced only by male fetal Leydig cells, whereas steroid hormones are also produced by the fetal adrenal gland (Anand-Ivell and Ivell 2014). Fetal urine is believed to be the primary source of steroid hormones in amniotic fluid from the second trimester onward (Schindler 1982). The source or sources of steroids in the amniotic fluid are unknown, but could include the umbilical cord or placenta (Schindler 1982). Progesterone and estradiol are produced in considerable amounts in the placenta, and may be partly transferred to the fetus (Pasqualini 2005). A recent *in vitro* study using human placental cells indicated that placental aromatase activity may be altered after exposure to PFOS (Gorrochategui et al. 2014). Thus, higher testosterone levels in amniotic fluid in our study population might be attributable to inhibited aromatase activity in the placenta. However, whereas androstendione and DHEAS concentrations were weakly correlated between second-trimester amniotic fluid and maternal serum samples from mothers expecting male offspring, testosterone concentrations were not correlated, suggesting limited maternal to fetal transfer of testosterone relative to other steroid hormones (van de Beek et al. 2004).

*In utero* exposure of rats to di(*n*-butyl) phthalate, a compound with antiandrogenic properties, caused reduced INSL3 expression in fetal Leydig cells and an increase in cryptorchidism, and INSL3 has been suggested as an endogenous marker of endocrine disruption (Anand-Ivell and Ivell 2014). A recent study from our group using the same study population as in the present study indicated that di(2-ethylhexyl) phthalate (DEHP) metabolites but not diisononyl phthalate (DiNP) metabolites were also related to decreased INSL3 level (Jensen et al. 2015). However, regarding PFOS exposure, a recent study indicated that fetal rat Leydig cell INSL3 production was unaltered after exposure to 20 mg/kg/day PFOS from gestational day 11 to 19 (Zhao et al. 2014). There may be species differences in effects of PFOS on INSL3 production, partly because of the much shorter half-life of PFOS in rats than in humans (Lau et al. 2004). In our study population, prenatal PFOS exposure was associated with lower INSL3 in amniotic fluid, but was not associated with cryptorchidism. Whether lower INSL3 levels can be associated with long-term effects on male reproductive health is not known. However, it is of interest to note that a recent follow-up study found lower sperm counts and increased LH and FSH levels in 19- to 21-year-old males at the highest tertile of PFOA exposure *in utero* compared with the lowest (Vested et al. 2013). In that study, PFOA and PFOS was estimated from maternal pregnancy serum samples. Although no association with PFOS was found in the Vested et al. (2013) study, PFOS and PFOA were strongly correlated in maternal serum samples (*r* = 0.73). We did not measure PFOA in the present study; thus it cannot be excluded that the associations with INSL3 may be related to an effect of PFOA rather than PFOS on INSL3. Future studies should evaluate whether low fetal INSL3 level is associated with long-term consequences for male reproductive health.

We measured PFOS, steroid hormones, and INSL3 in amniotic fluid for several reasons. Most amniotic fluid samples were taken in gestational weeks 15 to 17, close to the time window of male genital development in gestational weeks 8 to 15 (Scott et al. 2009). Contaminant concentrations in amniotic fluid during the first half of pregnancy, when the amniotic fluid is composed largely of exudates from fetal blood and fluids, are considered a suitable proxy measure of intrafetal contaminant levels (Beall et al. 2007).

Other studies on fetal PFOS exposures have used maternal serum or cord blood as a proxy for fetal exposure (Vested et al. 2013; Vesterholm et al. 2014). Although the presence in cord blood indicates that PFOS is transferred across the placenta to the fetus (Vesterholm et al. 2014), little is known about actual exposure level of the fetus, and we propose that measurement in amniotic fluid is the closest we can get to actual measurement of fetal exposure at the relevant time window of exposure (Jensen et al. 2012).

The strengths of the present study include the use of a large biobank of amniotic fluid samples linked with medical birth registries to produce a nested case–control sample of cryptorchidism and hypospadias cases selected purely based on clinical diagnoses that are unlikely to be related to the exposure. Although it might be speculated that socio-occupational class could be related to PFOS exposure and detection rate of cryptorchidism and hypospadias, we have previously shown that among Danish boys the time to detection of cryptorchidism is unrelated to socio-occupational class (Hougaard et al. 2014). However, our register-based ascertainment of cryptorchidism and hypospadias did not include milder forms of these outcomes that may spontaneously disappear during the first years of life, so we cannot exclude that PFOS exposure may be related to milder forms of genital malformations.

PFOS and hormone concentrations were measured concurrently in amniotic fluid samples, so we cannot confirm the temporal relation between PFOS exposure and the outcomes.

The analysis of associations between PFOS and hormone levels in the combined case and control groups may have produced biased estimates of associations in the source population, if associations in the case groups differed from the population as a whole. Our stratified and interaction analyses did not indicate major differences among the case and control groups, though sample sizes were small and estimates were imprecise, particularly for the hypospadias group. However, estimates based only on the controls, who should be representative of the overall population, were similar to the estimates based on the combined case and control groups (Table 3).

One may speculate that evaporation from stored samples or differential decay may explain some of the associations between hormone level and PFOS in amniotic fluid samples. However, PFOS, testosterone, and INSL3 levels were only weakly correlated with year of amniocentesis (*r*-values of 0.07, –0.06, and –0.05, respectively) and the estimated volume left in the biobank (*r*-values of 0.06, –0.06, and –0.02, respectively), suggesting that any influence of storage on the observed results would have been minor.

The concentration of PFOS in amniotic fluid samples in the present sample was somewhat higher than a previous U.S. study of pregnant women from New York, 2005–2008, where a median of 0.4 ng/mL PFOS was measured in amniotic fluid (Stein et al. 2012) compared with 1.1 ng/mL PFOS in the present study. This discrepancy probably stems from the phaseout of PFOS in the early 2000s, after our sample collection (1980–1996). Because of the higher concentration, we were therefore also able to detect PFOS in 98% of our samples compared with 32% in the recent U.S. study (Stein et al. 2012) despite a similar limit of detection and methodology used.

## Conclusions

Associations of PFOS with INSL3 and steroid hormone concentrations in amnionic fluid suggest that prenatal PFOS exposure may have affected fetal Leydig cell function in our study population. However, prenatal PFOS concentrations were not associated with cryptorchidism or hypospadias. Additional studies are needed to confirm associations between PFOS and fetal INSL3 and steroid hormone levels, evaluate potential mechanisms, and determine whether the estimated differences in fetal hormone levels are associated with long-term consequences for male reproductive health.
